# Brain network correlates of fatigue, depression, and anxiety in patients with Crohn’s Disease in different disease states

**DOI:** 10.1186/s12876-026-05097-6

**Published:** 2026-07-09

**Authors:** Anne Kerstin Thomann, Mike Michael Schmitgen, Jule Cara Stephan, Laura-Louise Knoedler, Philipp Arthur Thomann, Kristina Szabo, Matthias Philip Ebert, Wolfgang Reindl, Robert Christian Wolf

**Affiliations:** 1 Department of Medicine II, Medical Faculty Mannheim, Heidelberg University, Theodor-Kutzer-Ufer 1-3, Mannheim, 68167 Germany; 2 Center for Psychosocial Medicine, Department of General Psychiatry, Heidelberg University Hospital, Heidelberg University, Heidelberg, Germany; 3 Department of Psychiatry and Psychotherapy, SRH Clinic Karlsbad-Langensteinbach, Karlsbad, Germany; 4 Department of Neurology/Neuroimaging, Medical Faculty Mannheim, Mannheim Center for Translational Neurosciences (MCTN), University of Heidelberg, Mannheim, Germany

**Keywords:** Anxiety, Brain-Gut-Axis, Depression, Extraintestinal Symptoms, Fatigue, Inflammatory Bowel Diseases, Neurotransmitters, Resting-state brain activity

## Abstract

**Background:**

Symptoms of fatigue, depression or anxiety are frequent in Crohn’s Disease (CD) and may relate to disturbed brain-gut interactions. While more prevalent in active disease, these symptoms are also experienced by many individuals with CD during remission. Little is known about neural networks underlying such extraintestinal symptoms in CD and their relationship with the current disease state. Using a data fusion approach for functional MRI, this study investigated spatiotemporal markers of resting-state brain activity and associations with neurotransmitter systems and symptoms of fatigue, depression or anxiety in an active disease state or remission.

**Methods:**

We examined *n* = 71 patients with CD in an active disease state (aCD; *n* = 47) or in remission (rCD; *n* = 24) and healthy controls (HC; *n* = 35). All participants underwent resting-state fMRI, completed symptom assessments for fatigue, depression and anxiety, and provided stool samples for analysis of faecal calprotectin (fCal; aCD and rCD only). Joint independent component analysis (jICA) of two resting-state brain activity parameters (temporal and spatial features) identified neural networks exhibiting disease-state-dependent alterations. Network connectivity strength was correlated with symptoms of fatigue, depression, and anxiety, as well as fecal calprotectin (fCal). We further explored associations of the networks with neurotransmitter receptor maps.

**Results:**

JICA revealed three networks differentiating between disease states and/or between patients and controls. One network comprising affective orbitofrontal and temporal brain regions, exhibited reduced connectivity in active disease (HC vs. aCD: *p* = 0.003, *p*_FDR_ = 0.01; aCD vs. rCD: *p* < 0.001, *p*_FDR_ < 0.001) and was linked to serotonergic/dopaminergic transmission, fatigue, and fCal. Another network comprised sensorimotor brain regions and showed diminished connectivity in patients in remission (HC vs. rCD: *p* = 0.034, *p*_FDR_ = 0.06; aCD vs. rCD: *p* = 0.003, *p*_FDR_ = 0.01), correlating with depression, anxiety, and dopaminergic activity. The third network reflected the default-mode network topography and distinguished patients irrespective of disease status from controls (HC vs. aCD: *p* = 0.013, *p*_FDR_ = 0.01; HC vs. rCD: *p* = 0.037, *p*_FDR_ = 0.06), but showed no associations with symptoms.

**Conclusions:**

Resting-state brain network connectivity in patients with CD differed between active disease and remission, and was associated with symptoms of fatigue, depression, and anxiety. Alterations in sensorimotor networks were linked to depressive and anxiety symptoms, whereas affect-related networks were associated with fatigue. These observations suggest that distinct brain networks may contribute to specific neuropsychiatric symptom clusters in Crohn’s disease and underscore the role of brain–gut axis mechanisms in these manifestations.

**Graphical Abstract:**

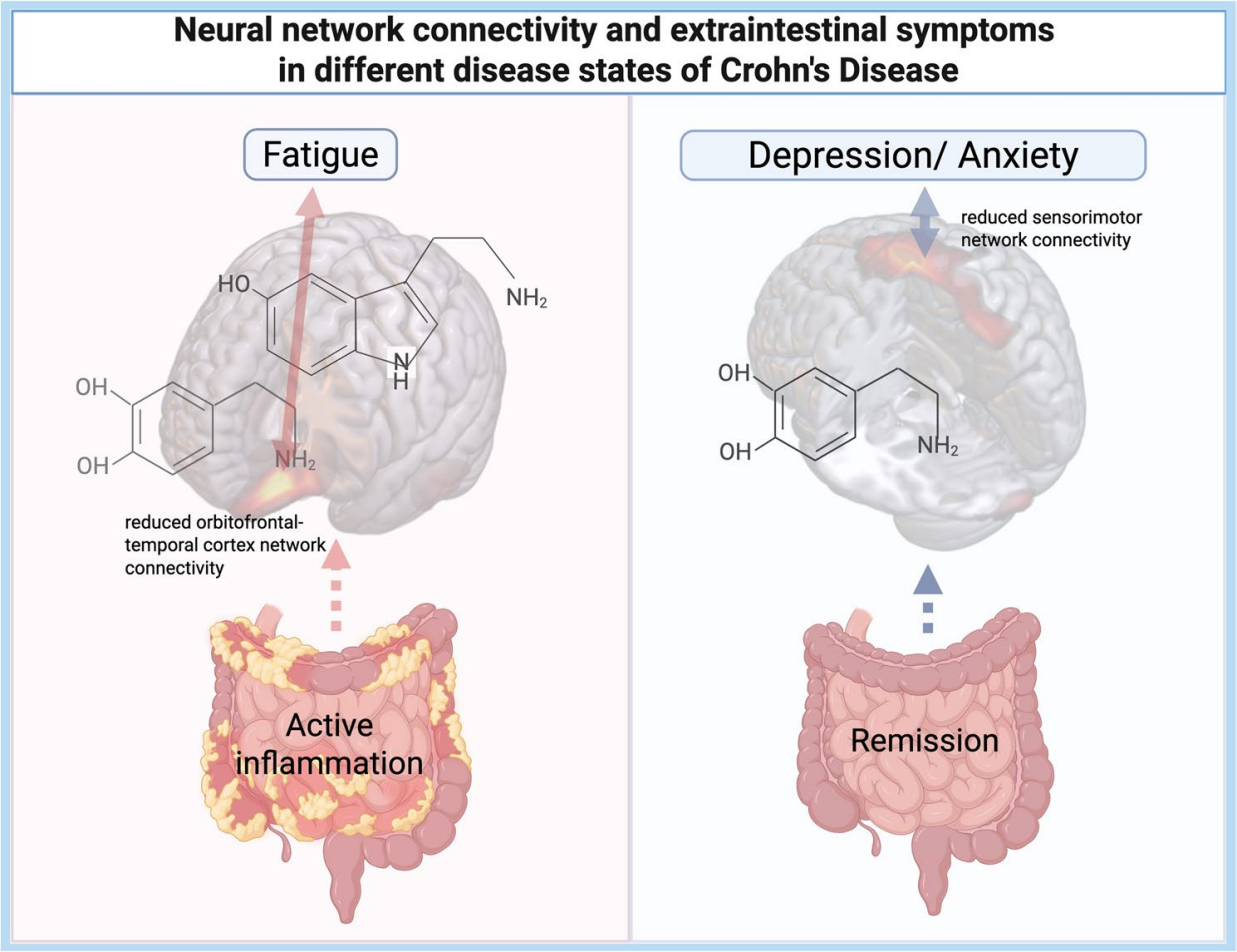

**Supplementary Information:**

The online version contains supplementary material available at 10.1186/s12876-026-05097-6.

## Background

Crohn’s Disease (CD) is a chronic inflammatory bowel disease (IBD) with a relapsing disease course. Inflammation can affect all parts of the gastrointestinal tract, causing symptoms such as abdominal pain or changes in bowel habits. However, the burden of CD often also encompasses symptoms outside the abdomen that are not directly related to inflammatory processes. Such “extraintestinal” symptoms comprise fatigue, anxiousness or depressive mood. In fact, the prevalence of anxiety and depression disorders in CD is two- to threefold higher than in the general population [1]. Fatigue affects up to 80% of patients during a flare, and up to half of all patients even in the absence of intestinal inflammation [2]. While all these symptoms are more common during active disease, they only resolve in a proportion of patients after successful treatment of the intestinal inflammation, suggesting differences in the pathomechanisms underlying these symptoms in remission vs. during a flare. Some neuropsychiatric symptoms in IBD are discussed to be a result of disturbed gut-brain-interactions caused by the chronic inflammatory burden, possibly mediated by the gut microbiota [3, 4]. Recent studies have also implicated changes in intestinal barrier [5] or blood-brain barrier [6] integrity in relation to dysregulated brain-gut-interactions.

Alterations of brain structure and function have been described in CD [7–12], partially related to symptoms of fatigue, depression or anxiety. While brain structure can be interpreted as a rather stable feature of an individual, brain function – often analyzed by magnetic resonance imaging (MRI) based examinations of fluctuations in blood oxygenation in the brain - can undergo rapid changes depending on current tasks that an individual is engaged in. Brain activity analysis of an individual at rest by examining resting-state functional MRI (rs-fMRI) allows the detection of intrinsic neural activity that is not influenced by a specific task and may give information about the functional connectivity of brain regions or networks.

A recent meta-analysis [11] included six rs-fMRI studies and detected activity changes in predominantly frontal brain areas in CD as compared to healthy controls.

It is unclear so far whether such brain activity changes are related to CD disease stage. The relapsing disease course of IBD with obvious differences not only in physical symptoms, but also in the emotional burden implies that brain activity changes in IBD could be disease state-dependent. Yet, until recently, studies examining brain function in IBD had almost exclusively included patients in remission. Also, few IBD-related studies considered symptoms of fatigue, depression or anxiety in brain activity analyses [13] and included those symptoms as covariates rather than variables of interest, potentially removing important brain-symptom relationships.

To our knowledge, putative relationships between resting-state brain activity and neuropsychiatric symptom load have been hardly addressed thus far. In a recent report, we identified disease-state dependent differences between active disease and remission predominantly in frontal regions, which were associated with fatigue and symptoms of anxiety and depression especially during remission [14]. Here, we expand these data by (i) providing an in-depth investigation of brain functional connectivity strength combined over temporal and spatial features, and by (ii) investigating neurochemical features of disease-dependent connectivity strength. To this end, we applied an MRI-based data fusion approach using joint independent component analysis (jICA), which enables the combined analysis of multiple measures of brain function [15, 16] and can reveal patterns that may not be detected with conventional methods. Specifically, unlike previous studies that focused on a single brain activity feature, we integrated both ALFF and ReHO to obtain a more comprehensive picture of functional connectivity strength. In addition, we explored potential links between these brain network patterns and underlying neurotransmitter systems using PET/SPECT receptor maps [17, 18]. Finally, we examined how network connectivity related to symptoms of fatigue, depression, and anxiety, as well as to the intestinal inflammatory marker faecal calprotectin (fCal).

## Methods

### Participants

The study was approved by the local ethics committee (2014–611 N-MA and 2017–601 N-MA) and conducted in accordance with the Declaration of Helsinki. All individuals provided written informed consent after a thorough explanation of the study objectives and procedures.

Patients with CD (PAT, *n* = 71) were recruited at the IBD outpatient unit at University Medical Center Mannheim, healthy control participants (HC, *n* = 35) were recruited via local advertising. Participants were excluded if they had MRI contraindications, a history of cancer, psychiatric or neurological disorders or current psychotropic medication. Other data of participants were also included in previous studies [7, 8, 14, 16].

Patients with active CD (aCD, *n* = 47) were in an active disease state, previously assessed in the department of medicine II, University Medical Center Mannheim. Disease activity was determined either by endoscopy with an SES-CD score of > 7 (*n* = 13), wall thickening with contrast enhancement in MRI enteroscopy (*n* = 10), bowel wall thickening >3 mm and increased color doppler sign (modified Limberg score of > 1) in sonography, (*n* = 14) and/or repeatedly elevated levels of fCal > 250 mg/kg (*n* = 24), and patients were already scheduled for a change of therapy or IBD-surgery before inclusion in the study. If brain MRI scans could not be obtained before the scheduled change of therapy or surgery, patients were excluded from the study. Patients with CD in remission (rCD, *n* = 24) were in stable remission without steroid use for at least six months and naïve to advanced IBD-therapies (biologicals or small molecules). Patient-reported disease activity was determined by Harvey Bradshaw-Index. Calprotectin levels were assessed in a period between 2 weeks before and 2 weeks after MRI scans were obtained.

### Questionnaires

Anxiety and depressive symptoms were assessed with the Hospital Anxiety and Depression Scale (HADS), a self-report screening tool widely used in different mental and somatic disorders that has been validated in IBD. Of its 14 items, two subscales with seven items each assess symptoms of depression and anxiety, respectively. Scores range from 0 to 21 points for each subscale, with higher scores indicating higher symptom load. We assessed fatigue with the Wurzburg Fatigue Inventory Multiple Sclerosis (WEIMuS), a 17-item self-report instrument originally developed for patients with multiple sclerosis. WEIMuS scores range from 0 to 68 points, with higher scores indicating more fatigue. All questionnaires were administered on the same day as MRI scans were obtained.

### MRI scanning

MRI scans were performed on a 3 T MAGNETOM Skyra whole body MR scanner (Siemens Medical Solutions, Erlangen, Germany) equipped with a 20-channel multi-array head-coil to collect whole-brain structural and functional scans in a darkened room at the Department of Neurology, Medical Faculty Mannheim, Heidelberg University.

#### Functional MRI

Functional MRI images were obtained using echo-planar imaging (EPI) in axial orientation (repetition time = 2210 ms, echo time = 23 ms, voxel size = 2.3 × 2.3 × 3.0 mm, 36 slices with sice thickness = 3.0 mm, recorded in interleaved ascending order). A total of 210 images were acquired within each scanning session. Participants were instructed to lie as still as possible, to relax with their eyes closed without falling asleep, and not to think about anything specific. Adherence to these instructions was verified by verbally contacting participants immediately after the resting-state scan (see supplementary materials for additional details).

### MRI data analysis

#### Data preprocessing

Data preprocessing was conducted using the Data Processing Assistant for Resting-State fMRI (DPARSF, https://rfmri.org/DPARSF), which is based on Statistical Parametric Mapping (SPM, https://www.fil.ion.ucl.ac.uk/spm/software/spm12/) and the toolbox for Data Processing & Analysis of Brain Imaging (DPABI, https://rfmri.org/DPABI) implemented in MATLAB (R2023b, the Math Works, Natick, MA). Preprocessing included discarding the first 10 images, slice timing, head motion correction, spatial normalization and smoothing. Spatial normalization (voxel size: 3 × 3 × 3 mm) was performed using the standard SPM12 EPI template (MNI-space). For spatial smoothing, we applied a 9 mm full-width half-maximum (FWHM) Gaussian Filter – before estimating temporal features or after estimating spatial features, respectively.

We calculated ALFF and ReHo as local measures of resting-state brain activity (temporal and spatial features, respectively) via DPARSF (see supplementary materials for details).

#### Synthesis of spatiotemporal features translated to network function

In this study, jICA on temporal and spatial features of resting-state brain activity was applied using the Fusion ICA Toolbox (FIT; version 2.0e; https://github.com/trendscenter/fit; last access: 02/06/2025) implemented in MATLAB 9.4.0 (R2023b). For both resting-state modalities, whole-brain maps were chosen as input for the analysis for each of the three groups (HC, rCD, and aCD). The resulting component maps reflect brain networks of shared information of the features included in the model. The key advantage of jICA over conventional descriptive approaches is its ability to simultaneously process multiple distinct information sources within a unified analysis framework [19].

For network visualization, the ICA data was reshaped back to a 3D-image, scaled to unit standard deviations (z), and a threshold of z > 3.0 was applied. Maps from the networks, showing differences between groups in ANCOVA models corrected for age and sex, described in the results section (see below) were overlaid onto an MNI normalized anatomical template. Anatomical denominations and stereotaxic coordinates were derived from clusters above a threshold of Z = 3.0 and a spatial extent threshold of ≥ 0.5 cm³. Networks showing a difference between HC, rCD, and/or aCD of at least *p* ≤ 0.1 were considered as networks of interest and entered statistical testing including correction for age and sex via ANCOVA models (see supplementary materials for additional details).

#### Neurochemical associations of resting-state brain activity

To link the observed networks of resting-state brain activity showing differences between groups with neurochemical properties, Spearman correlations of network maps with neurotransmitter/receptor maps were calculated via JuSpace toolbox (version 1.5; https://github.com/juryxy/JuSpace; last access 02/07/2025) [39]. The JuSpace toolbox facilitates correlations between imaging data and PET/SPECT-derived receptor maps based on a healthy reference population. Statistical inference was based on nominal significance level of *p* < 0.05, followed by Bonferroni-correction (see supplementary materials for additional details).

### Statistical analyses

Statistical analyses were performed via R (version 4.1.1) implemented in RStudio (version 2024.04.2). For differences in demographics and clinical parameters ANOVAs and two sample t-tests were calculated. Robust methods were used wherever appropriate (see Table 1). JICA identified five networks of interest, which entered statistical testing via ANCOVA models, using age and sex as covariates. Three of these networks showed significant differences in the ANCOVA models (uncorrected *p* < 0.05). Associations between FCS of these networks, neuropsychiatric symptom load, and fCal were investigated via Spearman correlations in PAT and a nominal significance level of *p* < 0.05 (uncorrected) was applied.

**Table 1:** Demographics and clinical parameters

	HC (mean)	SD	Min-Max	PAT (mean)	SD	Min-Max	rCD (mean)	SD	Min-Max	aCD (mean)	SD	Min-Max	Statistic (df) _HC vs. rCD vs. aCD_	*p*_HC vs. rCD vs. aCD_	Statistic (df) _HC vs. PAT_	*p*_HC vs. PAT_	Statistic (df) _HC vs. rCD_	*p*_HC vs. rCD_	Statistic (df) _HC vs. aCD_	*p*_HC vs. aCD_	Statistic (df) _rCD vs. acD_	*p*_rCD vs. aCD_
Sample size	35	-	-	71	-	-	24	-	-	47	-	-	-	-	-	-	-	-	-	-	-	-
Age	40.26	13.65	20–65	38.54	13.22	19–72	40.38	14.19	19–61	37.60	12.75	19–72	0.54 (2)^a^	0.59	1316.5^c^	0.62	412.5^c^	0.91	741^c^	0.45	492^c^	0.38
Sex (f/m)	25/10	-	-	39/32	-	-	16/8	-	-	23/24	-	-	4.76 (2)^b^	0.09	2.67 (1)^b^	0.10	0.15 (1)^b^	0.70	4.18 (1)^b^	**0.04**	2.02 (1)^b^	0.16
HADS anxiety score	3.69	2.70	0–11	6.17	3.64	0–15	5.29	3.99	0–13	6.62	3.41	0–15	7.73 (2)^a^	***< 0.001***	-3.57 (104)^d^	***< 0.001***	-1.72 (37.18)^e^	0.09	-4.20 (80)^d^	***< 0.001***	-1.46 (69)^d^	0.15
HADS depression score	2.00	2.65	0–10	5.46	4.38	0–18	3.86	3.58	0–14	6.17	4.55	1–18	15.30^f^	***< 0.001***	494^c^	***< 0.001***	235.5^c^	**0.05**	1292.5^c^	***< 0.001***	654.5^c^	**0.03**
WEIMuS total score	9.23	12.64	0–44	28.51	16.25	0–64	20.71	15.32	0–52	32.49	15.38	0–64	25.90 (2)^a^	***< 0.001***	406^c^	***< 0.001***	211^c^	***0.001***	1450^c^	***< 0.001***	-19.47 (69)^d^	***0.003***
WEIMuS cognitive score	4.60	6.98	0–24	13.21	7.98	0–29	9.67	7.39	0–25	15.02	7.72	0–29	19.98 (2)^a^	***< 0.001***	454.5^c^	***< 0.001***	210.5^c^	***0.001***	1401^c^	***< 0.001***	785^c^	***0.007***
WEIMuS somatic score	4.63	5.86	0–23	15.30	9.39	0–40	11.04	8.50	0–29	17.47	9.16	0–40	30.56^f^	***< 0.001***	406.5^c^	***< 0.001***	214^c^	***0.001***	1425.5^c^	***< 0.001***	-2.86 (69)^d^	***0.006***
fCal [mg/kg]	-	-	-	259.88	306.16	0-800	31.53	36.71	0-150	371.61	318.01	0-800	-	**-**	-	**-**	-	**-**	-	**-**	942^c^	***< 0.001***

*Abbreviations*: *HC* Healthy controls, *rCD* remitted group, *aCD* active group, *SD* Standard deviation, *df* degrees of freedom, *m* male, *f* female, *HADS* Hospital Anxiety and Depression Scale, *WEIMuS* Würzburg Fatigue Inventory in Multiple Sclerosis, *fCal* fecal calprotectin

^a^ ANOVA; ^b^ χ^2^; ^c^ Wilcoxon rank sum test; ^d^ Two Sample t-test; ^e^ Welch Two Sample t-test; ^f^ robust ANOVA with trimmed mean and 2000 bootstrap-samples

Due to missing values for HADS depression score, two HC and three rCD were omitted and one rCD was omitted due to missing value for fCal. Significant results in bold font, results surviving Bonferroni correction in italic font

## Results

### Study cohort

The final cohort consisted of *n* = 71 patients (*n* = 47 aCD and *n* = 24 rCD) and *n* = 35 HC. There was no age difference between the study groups and subgroups (HC, all patients, aCD, rCD). In terms of sex distribution, only HC and aCD differed, with more females in the control group (*p* < 0.04, χ² = 2.02 [df = 1]). We found expected differences in symptoms of fatigue, depression, anxiety, and fCal load: aCD showed the highest levels in all symptoms (mean values: WEIMuS total score = 32.49, WEIMuS cognitive score = 15.02, WEIMuS somatic score = 17.47, HADS depression score = 6.17, HADS anxiety score = 6.62, fCal = 371.61), followed by rCD (mean values: WEIMuS total score = 20.71, WEIMuS cognitive score = 9.67, WEIMuS somatic score = 11.04, HADS depression score = 3.86, HADS anxiety score = 5.92, fCal = 31.51), followed by HC (mean values: WEIMuS total score = 9.23, WEIMuS cognitive score = 4.60, WEIMuS somatic score = 4.63, HADS depression score = 2.00, HADS anxiety score = 3.69). Patients and HC differed significantly in fatigue and depression scores (all *p* < 0.001). ACD and rCD differed significantly in HADS depression score, WEIMuS scores and fCal (HADS depression score: *p* = 0.03, WEIMuS total fatigue score: *p* = 0.003, WEIMuS cognitive fatigue score: *p* = 0.007, WEIMuS somatic fatigue score *p* = 0.006, fCal: *p* < 0.001; see Tables 1 and 2 and Supplementary Table 1 for demographic information and patient disease characteristics).

**Table 2:** IBD characteristics of patients in remission and with active disease

IBD characteristics of patients	rCD (*n* = 24)	aCD (*n* = 47)
Disease duration in years, mean (SD)	16 (12.5)	13.7 (10.6)
Montreal classification, n (%)		
A1	6 (25%)	10 (21%)
A2	18 (75%)	34 (72%)
A3	-	3 (6%)
L1	11 (46%)	8 (17%)
L2	4 (17%)	8 (17%)
L3	6 (25%)	23 (49%)
L4	3 (13%)	7 (15%)
B1	12 (50%)	18 (38%)
B2	2 (8%)	16 (34%)
B3	11 (46%)	12 (26%)
Perianal disease	4 (17%)	15 (32%)
Prior exposure to advanced IBD therapies, n (%)		
Yes	0	18 (38%)
No	24 (100%)	29 (62%)
Prior IBD-related bowel resection, n (%)		
Yes	15 (62%)	21 (45%)
No	9 (38%)	26 (55%)
HBI, median (range)	1 (0–4)	7 (3–35)
Faecal calprotectin [mg/kg], mean (SD)	32 (36.71)	372 (318.01)

*Abbreviations*: *aCD* patients with active Crohn’s disease, *HBI* Harvey Bradshaw Index, *IBD* Inflammatory bowel disease, *rCD* patients with Crohn’s disease in remission, *SD* Standard deviation

### Synthesis of spatiotemporal brain activity features translated to network function

**Fig. 1:**
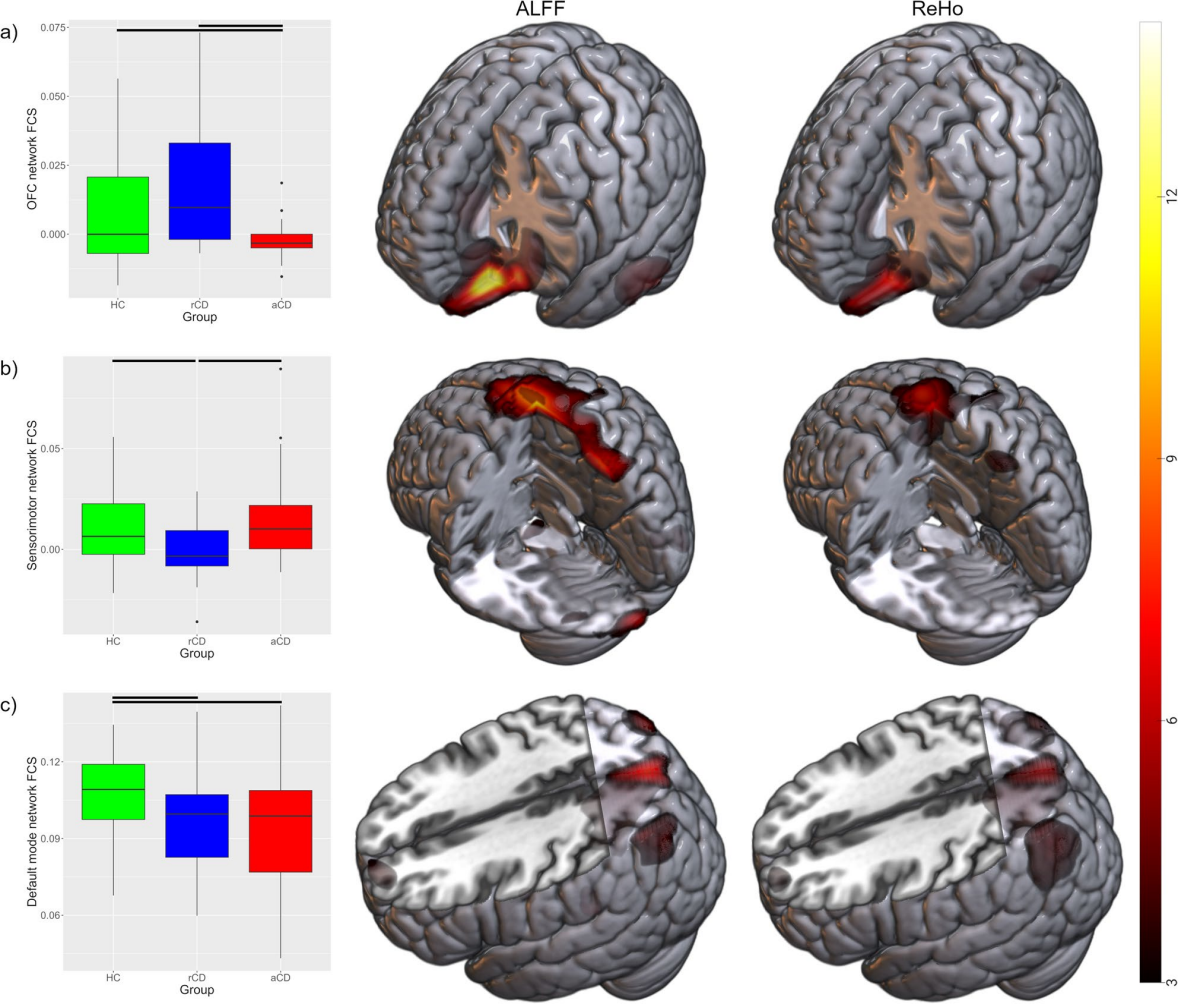
Functional connectivity strength (FCS) and localization of significant clusters of joint ICA components showing differences between groups. (**a**) Affective Orbitofrontal-temporal cortex network (**b**) Sensorimotor network (**c**) Component comprising the Default mode network; left: Boxplots of FCS per group, horizontal bars depict significant differences between groups; middle: Localization of significant clusters in temporal features of resting-state brain activation; right: Localization of significant clusters in spatial features of resting-state brain activation. Abbreviations: HC, Healthy controls; rCD, Remitted Crohn’s Disease patients; aCD, Active Crohn’s Disease Patients. Localization of significant clusters at Z > 3.0. Colorbar shows Z-values. This figure was created using R (https://www.r-project.org/, last visited 03/25/2025), MRIcroGL (https://www.nitrc.org/projects/mricrogl, last visited 03/26/2025) and GIMP (https://www.gimp.org/, last visited 03/25/2025)

Three of the five identified jICA networks of interest showed significant differences between groups. One network, mainly located in the orbitofrontal and inferior temporal cortex (OFC network, regions involved in affective processing and decision-making), differed between aCD and rCD (F(df = 1) = 32.01, *p* < 0.001, *p*_FDR_ < 0.001) as well as between HC and aCD (F(df = 1) = 9.75, *p* = 0.003, *p*_FDR_ = 0.01). One network, predominantly comprising sensorimotor regions (sensorimotor network, SMN) differed between HC and rCD (F(df = 1) = 4.72, *p* = 0.034, *p*_FDR_ = 0.06) as well as between aCD and rCD (F(df = 1) = 9.35, *p* = 0.003, *p*_FDR_ = 0.01). Finally, one network, mainly covering structures of the Default Mode Network (DMN) differed between HC and aCD (F(df = 1) = 6.53, *p* = 0.013, *p*_FDR_ = 0.01) as well as between HC and rCD (F(df = 1) = 4.58, *p* = 0.037, *p*_FDR_ = 0.06) (see Table 3; Fig. 1).

**Table 3:** Localization and extent of clusters within the functional networks showing differences between HC and patients. For the networks shown in Fig. 1, voxels > Z = 3.0 were converted from MNI to Talairach coordinates and coupled with the Talairach Daemon database to provide anatomical labels. Maximum Z-values and stereotaxic coordinates (x, y, z) are provided for each hemisphere (left = L, right = R). The volume of voxels in each area is provided in cubic centimeters (cc)

Brain area	Brodmann Area	Volume (cc) left/right	Z value (Talairach x, y, z) left/right	MNI (x, y, z) left/right
*ALFF OFC network (differing between aCD and rCD and between HC and aCD)*				
Rectal Gyrus	11	2.9/2.8	12.6 (-3, 16, -21)/11.5 (3, 25, -24)	(-3, 18, -24)/(3, 27, -27)
Inferior Frontal Gyrus	11, 47	3.0/0.4	10.0 (-18, 20, -16)/4.9 (15, 19, -19)	(-18, 21, -18)/(15, 21, -21)
Orbital Gyrus	11, 47	2.0/0.9	8.9 (-6, 31, -27)/5.7 (6, 37, -27)	(-6, 33, -30)/(6, 39, -30)
Fusiform Gyrus	20	1.0/1.0	8.3 (-50, -27, -24)/7.9 (50, -28, -26)	(-51, -27, -30)/(51, -27, -33)
Medial Frontal Gyrus	11, 25	1.8/1.2	8.3 (-3, 14, -18)/8.1 (3, 14, -18)	(-3, 15, -21)/(3, 15, -21)
Subcallosal Gyrus	13, 34, 47	1.0/0.2	8.2 (-18, 17, -13)/3.3 (12, 17, -13)	(-18, 18, -15)/(12, 18, -15)
Inferior Temporal Gyrus	20	1.0/0.9	5.8 (-56, -30, -21)/5.9 (48, -22, -29)	(-57, -30, -27)/(48, -21, -36)
*ReHo OFC network (differing between aCD and rCD and between HC and aCD)*				
Rectal Gyrus	11	2.6/2.2	7.8 (-6, 19, -26)/7.1 (3, 22, -26)	(-6, 21, -30)/(3, 24, -30)
Inferior Frontal Gyrus	11, 47	1.3/-	5.9 (-12, 14, -21)/-	(-12, 15, -24)/-
Orbital Gyrus	11, 47	1.2/1.0	5.6 (-6, 31, -27)/5.4 (6, 37, -27)	(-6, 33, -30)/(6, 39, -30)
Medial Frontal Gyrus	25	0.7/0.3	4.8 (-6, 17, -18)/4.1 (3, 14, -18)	(-6, 18, -21)/(3, 15, -21)
Declive	-	0.4/1.3	3.6 (-9, -74, -22)/4.7 (42, -77, -21)	(-9, -75, -30)/(42, -78, -30)
*ALFF SMN (differing between aCD and rCD and between HC and rCD)*				
Precuneus	7	0.6/1.3	5.3 (-3, -58, 61)/8.4 (3, -52, 61)	(-3, -63, 63)/(3, -57, 63)
Superior Frontal Gyrus	6	0.8/1.1	5.4 (-3, 0, 66)/7.6 (6, 9, 66)	(-3, -3, 72)/(6, 6, 72)
Lingual Gyrus	17, 18	1.8/-	6.2 (-12, -97, -8)/-	(-12, -99, -15)/-
Inferior Occipital Gyrus	17, 18, 19	0.6/0.2	4.5 (-21, -97, -10)/3.6 (39, -82, -11)	(-21, -99, -18)/(39, -84, -18)
Fusiform Gyrus	18, 19	0.4/1.0	3.8 (-21, -91, -11)/4.2 (42, -79, -11)	(-21, -93, -18)/(42, -81, -18)
*ReHo SMN (differing between aCD and rCD and between HC and rCD)*				
Superior Frontal Gyrus	6	0.7/1.3	6.3 (-3, 3, 66)/6.6 (6, 0, 66)	(-3, 0, 72)/(6, -3, 72)
Medial Frontal Gyrus	6	0.7/1.2	4.5 (-3, -8, 67)/5.4 (3, -8, 67)	(-3, -12, 72)/(3, -12, 72)
Precuneus	7	-/1.1	-/4.2 (6, -52, 61)	-/(6, -57, 63)
Cingulate Gyrus	24	0.6/0.6	4.1 (0, -1, 44)/4.0 (3, -4, 44)	(0, -3, 48)/(3, -6, 48)
Precentral Gyrus	4, 6	-/2.0	-/3.7 (33, -23, 62)	-/(33, -27, 66)
*ALFF DMN (differing between HC and rCD and between HC and aCD)*				
Precuneus	7, 19, 31	5.6/6.0	6.7 (-3, -61, 53)/7.9 (3, -61, 53)	(-3, -66, 54)/(3, -66, 54)
Superior Parietal Lobule	7	1.3/1.8	7.4 (-3, -64, 56)/7.1 (6, -64, 53)	(-3, -69, 57)/(6, -69, 54)
Posterior Cingulate	23, 29, 30, 31	1.9/2.2	5.6 (0, -49, 19)/5.2 (3, -51, 19)	(0, -51, 18)/(3, -54, 18)
Cingulate Gyrus	31	1.2/0.9	4.8 (0, -51, 27)/4.4 (3, -48, 27)	(0, -54, 27)/(3, -51, 27)
Inferior Parietal Lobule	7, 39, 40	1.7/1.0	4.4 (-42, -65, 47)/4.7 (42, -65, 47)	(-42, -69, 48)/(42, -69, 48)
Medial Frontal Gyrus	10	0.5/0.3	4.3 (-3, 61, -3)/3.5 (3, 61, -3)	(-3, 63, 0)/(3, 63, 0)
*ReHo DMN (differing between HC and rCD and between HC and aCD)*				
Precuneus	7, 19, 31, 39	9.8/9.3	6.0 (0, -51, 36)/5.6 (3, -54, 36)	(0, -54, 36)/(3, -57, 36)
Cingulate Gyrus	31	2.0/1.5	5.3 (0, -45, 35)/4.9 (3, -45, 33)	(0, -48, 36)/(3, -48, 33)
Superior Parietal Lobule	7	1.2/1.7	5.1 (-3, -64, 53)/4.8 (6, -64, 56)	(-3, -69, 54)/(6, -69, 57)
Inferior Parietal Lobule	7, 39, 40	3.4/1.5	4.8 (-42, -62, 45)/4.4 (42, -65, 45)	(-42, -66, 45)/(42, -69, 45)
Posterior Cingulate	23, 30, 31	1.2/1.2	4.8 (-3, -54, 25)/4.5 (3, -54, 25)	(-3, -57, 24)/(3, -57, 24)
Angular Gyrus	39	1.0/1.0	4.0 (-48, -62, 36)/3.8 (45, -65, 36)	(-48, -66, 36)/(45, -69, 36)
Supramarginal Gyrus	40	0.4/0.8	3.5 (-53, -57, 33)/3.9 (53, -57, 33)	(-54, -60, 33)/(54, -60, 33)
Middle Temporal Gyrus	39	0.3/0.8	3.3 (-50, -60, 28)/3.7 (50, -63, 28)	(-51, -63, 27)/(51, -66, 27)

*Abbreviations*: *ALFF* Amplitude of low-frequency fluctuations (temporal features), *DMN* Default mode network, *MNI* Montreal Neurological Institute, *OFC* Orbitofrontal-temporal cortex network, *ReHo* Regional Homogeneity (spatial features), *SMN* Sensorimotor network

### Neurochemical associations of resting-state brain activity

Spearman correlations of network maps showing significant group differences in the ANCOVA models (OFC network, SMN, and DMN) with neurotransmitter/receptor maps revealed a predominantly serotonergic, GABAergic, noradrenergic, and dopaminergic transmission in the OFC network, a mainly dopaminergic, noradrenergic, serotonergic, and cholinergic transmission in the SMN, and a predominantly serotonergic, dopaminergic, GABAergic, and cholinergic transmission in the DMN (see Table 4).

**Table 4:** Correlations between resting-state network activitys and neurotransmitter/receptor maps

Neurotransmitter/receptor	Fisher’s Z	*p*_exact_
*ALFF OFC network (differing between aCD and rCD and between HC and aCD)*		
5HT2a	-0.40	< 0.001
GABAa	-0.32	< 0.001
NAT	-0.46	< 0.001
*ReHo OFC network (differing between aCD and rCD and between HC and aCD)*		
5HT4	-0.38	< 0.001
D2	-0.34	< 0.001
*ALFF SMN (differing between aCD and rCD and between HC and rCD)*		
D2	-0.30	0.001
*ReHo SMN (differing between aCD and rCD and between HC and rCD)*		
DAT	0.31	< 0.001
FDOPA	0.33	< 0.001
NAT	0.40	< 0.001
SERT	0.30	0.001
VAChT	0.38	< 0.001
VAChT	0.37	< 0.001
VAChT	0.40	< 0.001
*ALFF DMN (differing between HC and rCD and between HC and aCD)*		
SERT	-0.30	< 0.001
*ReHo DMN (differing between HC and rCD and between HC and aCD)*		
5HT2a	0.54	< 0.001
DAT	-0.43	< 0.001
GABAa	0.48	< 0.001
SERT	-0.30	0.002
SERT	-0.43	< 0.001
VAChT	-0.36	< 0.001
VAChT	-0.40	< 0.001

Only results surviving Bonferroni-correction are displayed

*Abbreviations*: *5HT2a* Serotonine-receptor subtype 2a: Serotonine receptor subtype 4, *ALFF* Amplitude of low-frequency fluctuations (temporal features), *D2* Dopamine receptor 2, *DAT* Dopamine transporter, *DMN* Default mode network, *DOPA* Dihydroxyphenylalanine, *FDOPA* Fluorodopa, *GABAa* Gamma-amino butyric acid a, *NAT* Noradrenaline transporter, *OFC* Orbitofrontal-cingulate cortex, *ReHo* Regional Homogeneity (spatial features), *SERT* Serotonine transporter, *SMN* Sensorimotor network, *VAChT* Vesicular acetylcholine transporter

### Associations between network connectivity strength, symptoms of fatigue, depression or anxiety, and fCal

**Fig. 2:**
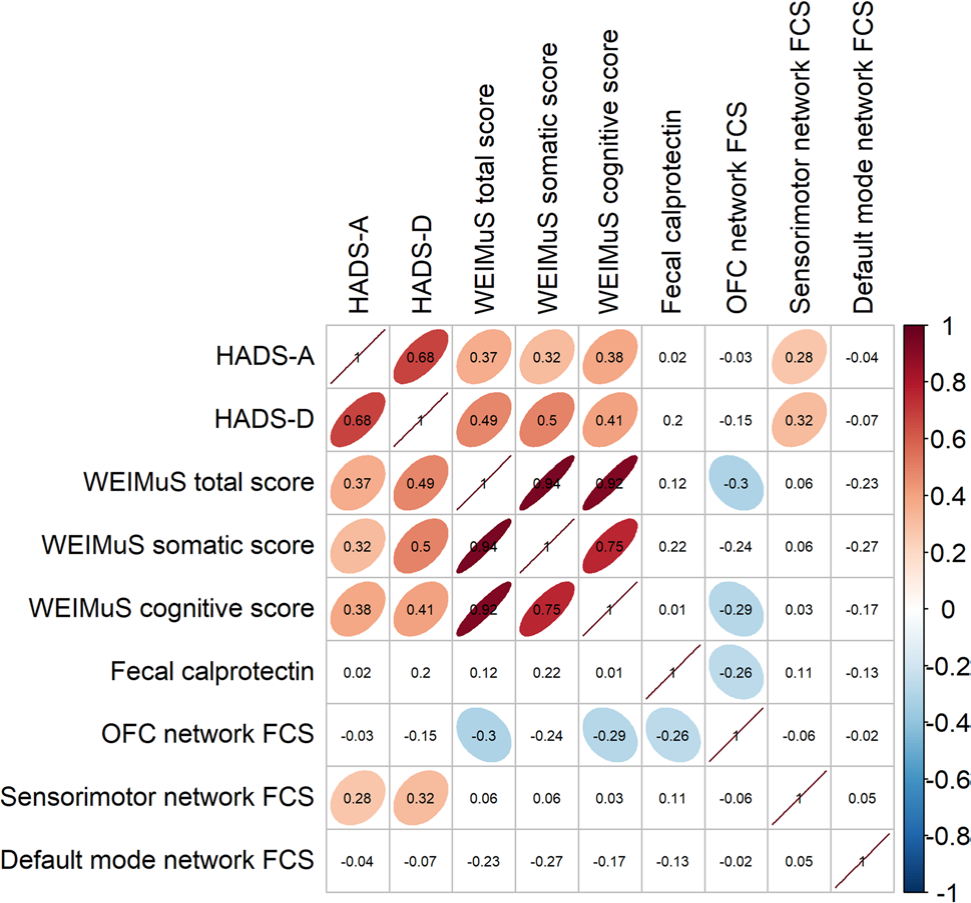
Correlation matrix of Spearman rank correlations between FCS, neuropsychiatric symptoms and fCal in patients with active CD and remission. Ellipses depict significant correlations at the *p* < 0.05 level (uncorrected). Numbers show Spearman’s *ρ*-values. Abbreviations: aCD, patients with active Crohn’s Disease ; rCD, patients with Crohn’s Disease in remission FCS, (Network) Functional connectivity strength; HADS, Hospital Anxiety and Depression Scale; HADS-A, HADS anxiety score; HADS-D, HADS depression score; OFC, orbitofrontal-temporal cortex network; WEIMuS, Wurzburg Fatigue Inventory Multiple Sclerosis. This figure was created using R and GIMP

Spearman correlations revealed negative associations between network connectivity of the OFC network and WEIMuS total score, WEIMuS cognitive score, and fCal (ρ = -0.30, ρ = -0.29, ρ = -0.26, respectively). Network connectivity of the SMN was positively associated with HADS-A and HADS-D scores (ρ = 0.28, ρ = 0.32, respectively). Of these correlations, only the association between connectivity strength of the SMN and HADS-D did not survive FDR-correction. Network connectivity of the DMN did not show significant correlations with any other tested parameters (see Fig. 2).

## Discussion

This study aimed to understand resting-state brain activity from a network and from a neurochemical perspective, by combining temporal and spatial resting-state brain activity in a data fusion approach and analyzing connectivity on a network level.

Using a data fusion approach combining two markers of resting-state brain activity (ALFF and ReHo), we identified three brain networks that differed between patients with active CD (aCD), patients in remission (rCD), and HC. An orbitofrontal (affective) network showed reduced connectivity in active disease compared with remission and controls. A sensorimotor network exhibited lower connectivity in patients in remission compared with both active disease and controls, while a network involving the DMN showed reduced connectivity in patients irrespective of disease state. These network patterns were also linked to clinical parameters: connectivity within the orbitofrontal network correlated with fatigue scores and fecal calprotectin (fCal), whereas the sensorimotor network was associated with symptoms of anxiety and depression. Finally, exploratory analyses suggested that these networks may be linked to distinct neurotransmitter systems, with serotonergic and dopaminergic contributions in the orbitofrontal network and dopaminergic involvement in the sensorimotor network.

This study substantially builds on our previous report [14] that provided first evidence of regionally confined disease-state dependent resting-state brain activity differences in CD. In particular, primarily frontal cortical resting-state brain activity changes in aCD, and associations between depressive symptoms and temporal features in the postcentral gyrus were detected, as well as between fatigue symptoms and temporal features in the subcallosal and spatial features in the medial orbital gyrus, in rCD.

Our finding of reduced connectivity in a frontotemporal network in patients with active disease compared to remission and controls, that is negatively correlated with fatigue, indicates a neural basis for the high fatigue prevalence especially during active disease. This may relate to feelings of exhaustion or frustration caused by the lack of energy during a flare, where much of an organism’s resources are needed for acute inflammatory processes, and resemble a state of sickness behavior including withdrawal from daily activities and the need for sleep. Connectivity strength in this network is lowest in aCD, at an intermediate level in HC and highest in rCD. This pattern possibly suggests that reduced connectivity in this network may represent a state characteristic of CD that normalizes with decreasing disease activity. This may also go along with the improvement of fatigue symptoms after successful treatment of a flare, which should be examined longitudinally. Fatigue symptoms in remission on the other hand may reflect a different issue that indicates a certain chronicity. This hypothesis is supported by previous findings from our group showing an association between fatigue and brain structure in remitted, but not active disease [8].

Anxiety and depressive symptoms in this study were associated with network connectivity in a sensorimotor network, with lower connectivity in rCD compared to both aCD and controls. Disease-state dependent differences of neural activity in motor pathways have also been previously described in CD [9]. The association between mood and anxiety symptoms and motor networks is in line with previous findings in depressive disorders [20, 21], and may relate to psychomotor retardation as a feature of depression. The fact that this network separated rCD from aCD and HC possibly suggests a chronically disturbed brain-gut-interaction as a plausible explanation for affective symptoms during remitted disease. The mechanisms underlying this disturbance are still unclear, but may include persistently impaired integrity of the intestinal [5, 22] as well as the blood-brain barrier [6] or microbiome alterations, which have been associated with both depression and fatigue in IBD [3].

Of note, fatigue and depressive symptoms show significant overlap and can influence each other negatively [23, 24]. In some patients, symptoms may be interpreted as fatigue but are actually a sign of an underlying depressive disorder [24]. On the other hand, fatigue symptoms impair quality of life and can negatively affect an individual’s mood, which may also result in a negative spiral of fatigue and depressive symptoms. Hence, fatigue and depressive symptoms can be difficult to disentangle. However, as they may need different therapeutic approaches, further information to distinguish between the two symptom complexes or to identify the primary issue possibly provoking or masking the other would be desirable. In this regard, our findings may suggest differences in the neural involvement of fatigue and depression, respectively.

This study further investigated the neurotransmitter systems underlying the identified neural networks associated with disease activity and symptoms of fatigue, depression, or anxiety in CD. The network most strongly linked to fatigue, which exhibited the lowest connectivity during active disease, was characterized by a predominance of serotonergic and dopaminergic transmission. Both serotonin and dopamine have been repeatedly implicated in the pathophysiology of fatigue [25], particularly in the context of exercise-induced fatigue, where disruptions in monoaminergic signaling contribute to reduced motivation, increased perceived effort, and central fatigue. Furthermore, recent neuroimaging research in multiple sclerosis, another disorder with prominent fatigue symptoms, has similarly demonstrated associations between fatigue severity and alterations in functional connectivity within dopamine- and serotonin-related networks [26]. These findings collectively point to monoaminergic dysfunction as a potential neurobiological mechanism underlying fatigue and highlight these pathways as promising targets for therapeutic intervention.

Additionally, we identified a distinct neural system - predominantly involving the sensorimotor network - that was associated with anxiety and depression in rCD and exhibited strong dopaminergic involvement. While serotonin and noradrenaline have traditionally been the primary neurotransmitters implicated in the pathophysiology of depression, accumulating evidence increasingly supports a role for dopamine in affective disorders [27]. Dopaminergic dysfunction has been linked to anhedonia, reduced motivation, and psychomotor retardation.

As a core feature of depression, psychomotor retardation encompasses a slowing of movement, speech, and cognitive processing and often manifests as reduced facial expressivity, delayed response times, and diminished motor activity [28]. This phenomenon is thought to arise from disruptions in cortico-striato-thalamo-cortical circuits, which play a crucial role in motor control, motivation, and goal-directed behavior. The sensorimotor network, a key component of these circuits, integrates motor planning and execution with affective and cognitive processes, making it a critical neural substrate for psychomotor function. In this study, the sensorimotor network exhibited altered functional connectivity in rCD and was predominantly linked to dopaminergic neurotransmission. This is consistent with evidence demonstrating that dopamine modulates motor output via the basal ganglia and prefrontal cortex, with dysfunction in these pathways contributing to the motor slowing observed in depression [29]. Reduced dopamine availability within the sensorimotor network may impair synaptic efficiency and neural signaling, leading to diminished motor drive and reinforcing the characteristic psychomotor retardation seen in depressive states. Moreover, dysfunction in this network may also contribute to a broader spectrum of motor-related symptoms in depression, including fatigue, reduced physical activity, and alterations in gait and posture. The identification of dopaminergic involvement in sensorimotor network abnormalities in rCD further underscores the relevance of this neurotransmitter system in the persistent depressive symptoms seen in IBD and suggests potential avenues for therapeutic interventions targeting dopamine-mediated motor-affective dysfunction. Congruously, dopaminergic transmission is also a core feature of several potent antidepressants. Our findings could motivate future interventions modulating these circuits and potentially offer therapeutic benefits for CD-associated neuropsychiatric symptoms.

We acknowledge potential limitations of this study, particularly the cross-sectional design, which allows the identification of associations between disease-related variables and neural network characteristics, but precludes conclusions regarding causality or changes across disease states. Longitudinal studies will be required to clarify how neural network properties and their associations with clinical outcomes evolve with disease activity. Such studies could also consider recently introduced methods addressing intestinal barrier or blood-brain-barrier integrity [6, 22, 30] to increase our pathophysiologic understanding of gut-brain disturbance.

A possible further limitation of our study may be that patients were recruited and stratified based on disease activity, but not neuropsychiatric symptom load, which led to rather low levels of clinically relevant symptoms in the study cohorts. In order to increase understanding of symptoms related to brain-gut-dysfunction, future research could explicitly address individuals with particularly high or low symptom burden or manifest psychiatric comorbidity. In this regard, the use of self-report assessment tools is another potential issue, as they may increase the risk of reporting bias. Furthermore, one of the screening tools (WEIMuS) was not formally validated in cohorts with IBD. It was chosen because of its clear structure, the possibility to distinguish between somatic and cognitive fatigue and the fact that no IBD-specific tool to assess fatigue was available in Germany at the time of study planning.

Furthermore, we chose to include only patients with CD, but not ulcerative colitis, to reduce heterogeneity within the study sample. Consequently, our findings apply specifically to CD and cannot be generalized to IBD as a whole. Finally, there are some notable differences in disease characteristics between the study groups (such as the difference in prior exposure to advanced therapies) that may have introduced an unknown bias in our results. This and the fact that several other parameters that could have influenced brain activity (e.g. other medication, sleep status, time of day, diet, hormonal disorders, other comorbidities etc.) have not been addressed in our study, need to be kept in mind when interpreting our findings.

## Conclusions

Our study identified distinct patterns of intrinsic brain activity in CD that varied by disease state and were partially associated with fatigue, depression, and anxiety. Notably, depression and anxiety were linked to reduced network connectivity in remission, whereas fatigue correlated with decreased connectivity in active disease. These findings suggest neurobiological differences not only between disease states but also between the predominant neuropsychiatric symptoms of CD. While both fatigue and affective symptoms are prevalent across all disease phases, our results suggest differences in the underlying neurobiological mechanisms. Specifically, fatigue appears to reflect transient alterations of brain activity associated with active inflammation, whereas persistent depressive symptoms in remission may indicate a chronic disruption of brain-gut interactions. If validated in longitudinal and/or interventional studies, these distinctions could inform personalized therapeutic strategies, including targeted psychotherapy or noninvasive neuromodulation, to address the differential mechanisms underlying fatigue and affective symptoms in CD.

## Supplementary Information

Supplementary Material 1

Supplementary Material 2

## Data Availability

All data needed to evaluate the conclusions in the paper are presented in the paper. Raw MRI data and parts of the clinical data can be provided by the corresponding authors upon reasonable request, pending scientific review and a completed material transfer agreement. Requests for underlying data should be submitted to the corresponding author.
