# Supplementary material for: Brain network correlates of fatigue, depression, and anxiety in patients with Crohn’s Disease in different disease states
Source: BMC Gastroenterol. 2026 Jul 9;26:438. doi: 10.1186/s12876-026-05097-6 (PMC13352982; doi:10.1186/s12876-026-05097-6)
Supplement: Supplementary file 1 — Supplementary Material 1 [file 12876_2026_5097_MOESM1_ESM.docx]

| **Parti-**  **cipant**  **#** | **Group** | **Age (years)** | **Sex (0=female)** | **Disease duration (years)** | **Montreal classification** | **Prior bowel resection** | **Harvey Bradshaw Index** | **Faecal Calprotectin** | **Definition of active disease (method)** | **HADS Anxiety** | **HADS Depression** | **WEIMuS total score** | **WEIMuS somatic fatigue** | **WEIMuS cognitive fatigue** |
| --- | --- | --- | --- | --- | --- | --- | --- | --- | --- | --- | --- | --- | --- | --- |
| 001 | HC | 25 | 0 | NA | NA | NA | NA | NA | NA | 7 | 9 | 26 | 11 | 15 |
| 002 | HC | 48 | 1 | NA | NA | NA | NA | NA | NA | 0 | 2 | 0 | 0 | 0 |
| 003 | HC | 28 | 0 | NA | NA | NA | NA | NA | NA | 7 | 1 | 44 | 23 | 21 |
| 004 | HC | 24 | 0 | NA | NA | NA | NA | NA | NA | 4 | 0 | 0 | 0 | 0 |
| 005 | HC | 33 | 0 | NA | NA | NA | NA | NA | NA | 6 | 1 | 3 | 3 | 0 |
| 006 | HC | 53 | 0 | NA | NA | NA | NA | NA | NA | 11 | 10 | 28 | 12 | 16 |
| 007 | HC | 30 | 1 | NA | NA | NA | NA | NA | NA | 4 | 0 | 0 | 0 | 0 |
| 008 | HC | 40 | 0 | NA | NA | NA | NA | NA | NA | 2 | 1 | 0 | 0 | 0 |
| 009 | HC | 65 | 0 | NA | NA | NA | NA | NA | NA | 5 | 2 | 0 | 0 | 0 |
| 010 | HC | 44 | 1 | NA | NA | NA | NA | NA | NA | 4 | 0 | 5 | 1 | 4 |
| 011 | HC | 51 | 0 | NA | NA | NA | NA | NA | NA | 3 | NA | 9 | 8 | 1 |
| 012 | HC | 55 | 0 | NA | NA | NA | NA | NA | NA | 4 | NA | 12 | 6 | 6 |
| 013 | HC | 60 | 1 | NA | NA | NA | NA | NA | NA | 5 | 1 | 42 | 18 | 24 |
| 014 | HC | 20 | 0 | NA | NA | NA | NA | NA | NA | 3 | 3 | 3 | 2 | 1 |
| 015 | HC | 36 | 0 | NA | NA | NA | NA | NA | NA | 2 | 3 | 9 | 6 | 3 |
| 016 | HC | 38 | 1 | NA | NA | NA | NA | NA | NA | 8 | 8 | 19 | 9 | 10 |
| 017 | HC | 51 | 1 | NA | NA | NA | NA | NA | NA | 0 | 0 | 0 | 0 | 0 |
| 018 | HC | 30 | 1 | NA | NA | NA | NA | NA | NA | 0 | 0 | 2 | 1 | 1 |
| 019 | HC | 48 | 0 | NA | NA | NA | NA | NA | NA | 8 | 4 | 8 | 6 | 2 |
| 020 | HC | 32 | 0 | NA | NA | NA | NA | NA | NA | 0 | 0 | 1 | 1 | 0 |
| 021 | HC | 27 | 1 | NA | NA | NA | NA | NA | NA | 1 | 0 | 3 | 2 | 1 |
| 022 | HC | 26 | 0 | NA | NA | NA | NA | NA | NA | 3 | 2 | 39 | 17 | 22 |
| 023 | HC | 61 | 0 | NA | NA | NA | NA | NA | NA | 5 | 3 | 13 | 7 | 6 |
| 024 | HC | 38 | 0 | NA | NA | NA | NA | NA | NA | 3 | 0 | 10 | 6 | 4 |
| 025 | HC | 30 | 0 | NA | NA | NA | NA | NA | NA | 2 | 0 | 5 | 3 | 2 |
| 026 | HC | 26 | 1 | NA | NA | NA | NA | NA | NA | 2 | 0 | 1 | 1 | 0 |
| 027 | HC | 27 | 0 | NA | NA | NA | NA | NA | NA | 8 | 2 | 20 | 10 | 10 |
| 028 | HC | 21 | 0 | NA | NA | NA | NA | NA | NA | 0 | 0 | 5 | 2 | 3 |
| 029 | HC | 57 | 0 | NA | NA | NA | NA | NA | NA | 4 | 2 | 0 | 0 | 0 |
| 030 | HC | 55 | 0 | NA | NA | NA | NA | NA | NA | 4 | 1 | 0 | 0 | 0 |
| 031 | HC | 57 | 0 | NA | NA | NA | NA | NA | NA | 3 | 1 | 0 | 0 | 0 |
| 032 | HC | 23 | 0 | NA | NA | NA | NA | NA | NA | 1 | 1 | 1 | 1 | 0 |
| 033 | HC | 55 | 0 | NA | NA | NA | NA | NA | NA | 5 | 6 | 15 | 6 | 9 |
| 034 | HC | 40 | 1 | NA | NA | NA | NA | NA | NA | 4 | 2 | 0 | 0 | 0 |
| 035 | HC | 55 | 0 | NA | NA | NA | NA | NA | NA | 1 | 1 | 0 | 0 | 0 |
| 036 | rCD | 20 | 1 | 6 | A1L2B1 | no | 1 | 26 | NA | 0 | 0 | 18 | 10 | 8 |
| 037 | rCD | 42 | 0 | 26 | A2L2B2 | yes | 3 | 47 | NA | 3 | NA | 0 | 0 | 0 |
| 038 | rCD | 46 | 0 | 8 | A2L1B1 | yes | 2 | 75 | NA | 2 | 4 | 45 | 22 | 23 |
| 039 | rCD | 31 | 1 | 16 | A1L4B3p | no | 1 | 7 | NA | 7 | 7 | 16 | 12 | 4 |
| 040 | rCD | 29 | 0 | 7 | A2L3B3 | yes | 0 | 15 | NA | 8 | 5 | 7 | 4 | 3 |
| 041 | rCD | 38 | 0 | 5 | A2L3B3 | yes | 2 | 11 | NA | 9 | 5 | 26 | 13 | 13 |
| 042 | rCD | 24 | 0 | 4 | A2L1B3 | yes | 3 | 8 | NA | 10 | 5 | 29 | 14 | 15 |
| 043 | rCD | 37 | 0 | 6 | A2L1B1 | no | 0 | 29 | NA | 13 | 8 | 13 | 7 | 6 |
| 044 | rCD | 59 | 0 | 32 | A2L1B1 | yes | 0 | 14 | NA | 5 | 0 | 0 | 0 | 0 |
| 045 | rCD | 58 | 1 | 55 | A1L1B1 | yes | 1 | 107 | NA | 2 | 3 | 13 | 9 | 4 |
| 046 | rCD | 57 | 0 | 26 | A2L2B1 | no | 1 | 43 | NA | 1 | NA | 22 | 9 | 13 |
| 047 | rCD | 26 | 0 | 15 | A1L4B3p | no | 2 | 9 | NA | 4 | 1 | 12 | 5 | 7 |
| 048 | rCD | 41 | 1 | 19 | A2L1B2 | yes | 2 | 150 | NA | 10 | 14 | 8 | 3 | 5 |
| 049 | rCD | 55 | 0 | 18 | A2L1B1 | yes | 0 | 8 | NA | 9 | NA | 33 | 22 | 11 |
| 050 | rCD | 53 | 0 | 16 | A2L1B1 | no | 1 | 0 | NA | 4 | 8 | 52 | 29 | 23 |
| 051 | rCD | 28 | 0 | 11 | A2L1B3 | yes | 0 | 46 | NA | 0 | 1 | 18 | 6 | 12 |
| 052 | rCD | 31 | 1 | 12 | A2L2B1 | no | 1 | 16 | NA | 2 | 1 | 5 | 2 | 3 |
| 053 | rCD | 19 | 0 | 1 | A1L1B3 | yes | 3 | 0 | NA | 4 | 4 | 33 | 19 | 14 |
| 054 | rCD | 20 | 1 | 4 | A2L4B1 | no | 2 | 52 | NA | 3 | 2 | 25 | 14 | 11 |
| 055 | rCD | 61 | 0 | 34 | A2L3B3p | yes | 1 | 8 | NA | 5 | 0 | 40 | 20 | 20 |
| 056 | rCD | 37 | 1 | 22 | A1L3B1 | yes | 1 | 17 | NA | 0 | 0 | 5 | 1 | 4 |
| 057 | rCD | 42 | 0 | 6 | A2L3B3p | yes | 1 | 34 | NA | 4 | 1 | 19 | 15 | 4 |
| 058 | rCD | 61 | 0 | 9 | A2L1B3 | yes | 4 | 5 | NA | 13 | 6 | 51 | 26 | 25 |
| 059 | rCD | 54 | 1 | 26 | A2L3B1 | no | 4 | NA | NA | 9 | 6 | 7 | 3 | 4 |
| 060 | aCD | 23 | 1 | 5 | A2L1B2p | yes | 8 | 29 | MRI | 2 | 1 | 8 | 5 | 3 |
| 061 | aCD | 51 | 1 | 12 | A2L3B2 | yes | 6 | 800 | MRI+fCal | 1 | 2 | 27 | 14 | 13 |
| 062 | aCD | 26 | 0 | 5 | A2L3B2p | no | 3 | 643 | MRI+fCal | 4 | 4 | 28 | 15 | 13 |
| 063 | aCD | 31 | 0 | 15 | A1L3B2 | yes | 3 | 224 | MRI | 13 | 7 | 24 | 10 | 14 |
| 064 | aCD | 47 | 1 | 26 | A2L3B2p | yes | 3 | 17 | MRI + Endoscopy | 6 | 2 | 29 | 12 | 17 |
| 065 | aCD | 29 | 1 | 3 | A2L4B1p | yes | 7 | 410 | fCal | 7 | 3 | 35 | 21 | 14 |
| 066 | aCD | 58 | 0 | 12 | A3L3B3p | yes | 35 | 57 | MRI + Endoscopy | 14 | 16 | 64 | 40 | 24 |
| 067 | aCD | 67 | 1 | 2 | A3L1B1 | yes | 7 | 800 | Sonography + fCal | 5 | 4 | 40 | 25 | 15 |
| 068 | aCD | 20 | 0 | 5 | A1L2B1 | no | 6 | 400 | fCal | 7 | 3 | 15 | 9 | 6 |
| 069 | aCD | 25 | 1 | 2 | A2L2B1 | no | 20 | 800 | fCal + Endoscopy | 9 | 16 | 40 | 29 | 11 |
| 070 | aCD | 60 | 0 | 42 | A2L3B3p | yes | 13 | 46 | Sonography, MRI | 6 | 2 | 50 | 26 | 24 |
| 071 | aCD | 49 | 0 | 25 | A2L2B3p | yes | 9 | 381 | fCal | 6 | 7 | 43 | 23 | 20 |
| 072 | aCD | 26 | 0 | 5 | A1/2L1B3 | yes | 15 | 178 | Sonography | 6 | 4 | 33 | 17 | 16 |
| 073 | aCD | 32 | 0 | 3 | A2L2B1 | no | 5 | 233 | Endo | 5 | 1 | 15 | 9 | 6 |
| 074 | aCD | 27 | 1 | 7 | A2L4B1 | no | 10 | 773 | fCal | 6 | 4 | 49 | 21 | 28 |
| 075 | aCD | 37 | 1 | 1 | A2L2B1 | no | 15 | 30 | Endoscopy | 6 | 8 | 33 | 22 | 11 |
| 076 | aCD | 23 | 1 | 11 | A1L3B1 | no | 15 | 800 | fCal | 1 | 7 | 50 | 31 | 19 |
| 077 | aCD | 37 | 1 | 11 | A2L3B1 | yes | 7 | 786 | MRI + fCal | 10 | 8 | 21 | 11 | 10 |
| 078 | aCD | 32 | 1 | 8 | A2L2B3p | no | 8 | 770 | fCal | 4 | 6 | 23 | 18 | 5 |
| 079 | aCD | 37 | 0 | 21 | A1L3B1 | no | 7 | 534 | Sonography, MRI, fCal | 8 | 3 | 46 | 30 | 16 |
| 080 | aCD | 35 | 0 | 21 | A1L4B1 | yes | 7 | 162 | Sonography | 5 | 2 | 15 | 6 | 9 |
| 081 | aCD | 34 | 0 | 16 | A2L4B2 | no | 7 | 608 | fCal | 15 | 7 | 46 | 24 | 22 |
| 082 | aCD | 25 | 1 | 1 | A2L4B2 | no | 6 | 104 | Endoscopy | 3 | 2 | 12 | 9 | 3 |
| 083 | aCD | 35 | 1 | 10 | A2L2B2 | yes | 12 | 407 | Endoscopy + fC | 7 | 18 | 15 | 6 | 9 |
| 084 | aCD | 19 | 0 | 9 | A1L4B1 | no | 5 | 35 | Sonography | 6 | 5 | 10 | 3 | 7 |
| 085 | aCD | 43 | 0 | 23 | A2L1B2p | no | 6 | 100 | Endoscopy | 6 | 3 | 5 | 3 | 2 |
| 086 | aCD | 39 | 0 | 7 | A2L1B1 | no | 6 | 150 | Sonography | 3 | 1 | 26 | 11 | 15 |
| 087 | aCD | 34 | 1 | 13 | A2L3B3 | yes | 10 | 134 | Endoscopy + MRI | 3 | 2 | 19 | 7 | 12 |
| 088 | aCD | 57 | 1 | 34 | A1L3B2 | yes | 14 | 527 | Endoscopy + fCal | 12 | 9 | 46 | 21 | 25 |
| 089 | aCD | 34 | 1 | 16 | A2L4B2 | no | 6 | 800 | Sonography + fCal | 9 | 8 | 44 | 31 | 13 |
| 090 | aCD | 72 | 0 | 36 | A2L3B2 | yes | 5 | 453 | fCal | 9 | 7 | 55 | 26 | 29 |
| 091 | aCD | 45 | 1 | 19 | A2L3B2 | yes | 3 | 55 | Endoscopy | 6 | 3 | 14 | 5 | 9 |
| 092 | aCD | 32 | 1 | 19 | A1L1B3p | yes | 2 | 707 | fCal | 0 | 3 | 21 | 19 | 2 |
| 093 | aCD | 45 | 0 | 23 | A2L3B3p | yes | 7 | 55 | Sonography | 8 | 6 | 49 | 26 | 23 |
| 094 | aCD | 56 | 1 | 40 | A1L3B3p | no | 6 | 40 | Sonography | 7 | 4 | 25 | 8 | 17 |
| 095 | aCD | 25 | 0 | 7 | A2L3B1 | no | 4 | 28 | Sonography | 3 | 2 | 29 | 12 | 17 |
| 096 | aCD | 57 | 0 | 28 | A2L1B2 | yes | 26 | 12 | Endoscopy | 4 | 7 | 48 | 23 | 25 |
| 097 | aCD | 35 | 0 | 2 | A2L3B1 | no | NA | 425 | fCal | 4 | 7 | 37 | 18 | 19 |
| 098 | aCD | 31 | 1 | 11 | A2L3B3p | no | 4 | 800 | fCal | 6 | 18 | 28 | 19 | 9 |
| 099 | aCD | 53 | 0 | 3 | A3L2B2 | no | 3 | 800 | Endo + fCal | 4 | 2 | 0 | 0 | 0 |
| 100 | aCD | 30 | 1 | 6 | A2L3B1 | no | 10 | 800 | fCal | 12 | 11 | 51 | 27 | 24 |
| 101 | aCD | 34 | 1 | 12 | A2L3B1 | no | 7 | 56 | Sonography | 10 | 10 | 50 | 22 | 28 |
| 102 | aCD | 25 | 1 | 3 | A2L2B1p | no | 3 | 11 | Sonography | 11 | 8 | 31 | 13 | 18 |
| 103 | aCD | 36 | 1 | 15 | A2L3B3p | no | 10 | 678 | fCal | 6 | 2 | 38 | 20 | 18 |
| 104 | aCD | 41 | 0 | 23 | A2L3B3 | no | 8 | 0 | Sonography | 8 | 11 | 52 | 29 | 23 |
| 105 | aCD | 28 | 0 | 10 | A2L1B3 | yes | 12 | 9 | MRI | 11 | 11 | 47 | 23 | 24 |
| 106 | aCD | 30 | 0 | 14 | A2L3B2 | no | 14 | 800 | fCal | 7 | 13 | 41 | 22 | 19 |
