# Supplementary material for: Brain network correlates of fatigue, depression, and anxiety in patients with Crohn’s Disease in different disease states
Source: BMC Gastroenterol. 2026 Jul 9;26:438. doi: 10.1186/s12876-026-05097-6 (PMC13352982; doi:10.1186/s12876-026-05097-6)
Supplement: Supplementary file 2 — Supplementary Material 2 [file 12876_2026_5097_MOESM2_ESM.docx]

Supplementary materials for:

**Brain network correlates of fatigue, depression, and anxiety in patients with Crohn’s Disease in different disease states**

by:

Anne Kerstin Thomann^1*^, Mike Michael Schmitgen^2^, Jule Cara Stephan^1^, Laura-Louise Knoedler^1^, Philipp Arthur Thomann^3^, Kristina Szabo^4^, Matthias Philip Ebert^1^, Wolfgang Reindl^1^, Robert Christian Wolf^2^

^1^ Department of Medicine II, Medical Faculty Mannheim, Heidelberg University, Mannheim, Germany

^2^ Center for Psychosocial Medicine, Department of General Psychiatry, Heidelberg University Hospital, Heidelberg University, Heidelberg, Germany

^3^ Department of Psychiatry and Psychotherapy, SRH Clinic Karlsbad-Langensteinbach, Karlsbad, Germany

^4^ Department of Neurology/Neuroimaging, Medical Faculty Mannheim, Mannheim Center for Translational Neurosciences (MCTN), University of Heidelberg, Mannheim, Germany

**Methods**

*Functional MRI*

T2*-weighted images were obtained using echo-planar imaging (EPI) in an axial orientation (repetition time = 2210 ms, echo time = 23 ms, field of view = 220 × 220 mm, matrix size = 96 × 96 voxels, voxel size = 2.3 × 2.3 × 3.0 mm, flip angle = 90°, bandwidth = 1270 Hz/pixel, parallel acquisition technique GRAPPA acceleration factor 2, 36 slices with slice thickness = 3.0 mm, gap between slices = 0 mm, recorded in interleaved ascending order). A total of 210 images were acquired within each scanning session. Participants were instructed to lie as still as possible, to relax with their eyes closed without falling asleep, and not to think about anything specific. Adherence to these instructions was verified by verbally contacting participants immediately after the resting-state scan. No task-based scanning was included in the protocol.

## MRI data analysis

### Data preprocessing

Data preprocessing was conducted using the Data Processing Assistant for Resting-State fMRI (DPARSF, <https://rfmri.org/DPARSF>), which is based on Statistical Parametric Mapping (SPM, <https://www.fil.ion.ucl.ac.uk/spm/software/spm12/>) and the toolbox for Data Processing & Analysis of Brain Imaging (DPABI, <https://rfmri.org/DPABI>) implemented in MATLAB (R2023b, the Math Works, Natick, MA). Preprocessing included discarding the first 10 images (due to magnetization issues and participant acclimatization during the first images shot), slice timing, head motion correction, spatial normalization and smoothing. Spatial normalization (voxel size: 3 × 3 × 3 mm) was performed using the standard SPM12 EPI template (MNI-space). For spatial smoothing, we applied a 9 mm full-width half-maximum (FWHM) Gaussian Filter – before estimating temporal features or after estimating spatial features, respectively.

We calculated ALFF and ReHo as local measures of resting-state brain activity (temporal and spatial features, respectively) via DPARSF. ALFF detects the regional intensity of spontaneous fluctuations in the blood-oxygen level-dependent (BOLD) signal, reflecting spontaneous neural activity of specific regions and physiological states of the brain [1, 2]. ReHo measures synchronicity of time series between a given voxel and each of its neighbouring voxels [3-5], characterizing spatial organisation of brain function and its relationship with other network-level features [6]. We used the DPARSF to calculate temporal and spatial features with the following parameters: preprocessed images were bandpass filtered at 0.01-0.1 Hz. Kendall’s coefficient of concordance of time series was calculated to measure the spatial features of a given voxel with its 27 nearest neighbours on a voxel-by-voxel manner and the spatial features of each voxel was divided by the global mean the spatial features to gain standardized conditions. Afterwards, we used a 9 mm FWHM Gaussian filter to smooth the spatial features signals.

### Synthesis of spatiotemporal features translated to network function

JICA extracts maximally spatially independent maps linked by a shared loading parameter through five steps. First, features are computed for each individual. Second, features are normalized using the average sum of squares for each feature. Third, data from each feature are stacked across columns, with rows representing subjects. Fourth, Principal Component Analysis (PCA) reduces data dimensionality from subjects to components. Fifth, spatially independent components are extracted, each sharing a common loading parameter across the features included in the jICA analysis (here: temporal and spatial features of resting-state brain activation). The resulting component maps reflect brain networks of shared information of the features included in the model. The key advantage of jICA over conventional descriptive approaches is its ability to simultaneously process multiple distinct information sources within a unified analysis framework [7-13].

In this study, jICA on temporal and spatial features of resting-state brain activity was applied using the Fusion ICA Toolbox (FIT; version 2.0e; https://github.com/trendscenter/fit; last access: 02/06/2025) implemented in MATLAB 9.4.0 (R2023b). For both resting-state modalities, whole-brain maps were chosen as input for the analysis for each of the three groups (HC, rCD, and aCD). Estimation of the number of networks using minimum description length proposed 12 independent networks. Therefore, 12 networks were extracted from the data using the Infomax algorithm and ICASSO [14] was used to assess results after running the approach 20 times to ensure consistency of the networks. Apart from the parameters mentioned here, settings were kept at FIT default values.

For network visualization, the source matrix was reshaped back to a 3D-image, scaled to unit standard deviations (z), and a threshold of z > 3.0 was applied. Maps from the networks showing differences between groups in ANCOVA models corrected for age and sex, described in the results section were overlaid onto an MNI normalized anatomical template. Anatomical denominations and stereotaxic coordinates were derived from clusters above a threshold of z = 3.0 by using the “Write Talairach Table” function implemented in FIT. Significant clusters within the detected networks are reported using a spatial extent threshold of ≥ 0.5 cm³. Networks showing a difference in functional connectivity strength between HC, rCD, and/or aCD at a significance level of at least *p* ≤ 0.1 in FIT (independent *t*-tests) were considered as networks of interest (five of the 12 extracted networks were identified as networks of interest) and entered statistical testing including correction for age and sex via ANCOVA models. The liberal α-threshold of *p* ≤ 0.1 was chosen at the first step to prevent β-errors (false negatives) in terms of networks showing a *p* value of < 0.05 after correction for age and sex via ANCOVA models (three of the five networks of interest showed group differences at an uncorrected level of *p* < 0.05).

### Neurochemical associations of resting-state brain activity

### To link the observed networks of resting-state brain activity showing differences between groups with neurochemical properties, cross-modal Spearman correlations of network maps with neurotransmitter/receptor maps were calculated via JuSpace toolbox (version 1.5; <https://github.com/juryxy/JuSpace>; last access 02/07/2025) [15]. The JuSpace toolbox facilitates correlations between imaging data and PET/SPECT-derived receptor maps based on a healthy reference population. Using the JuSpace algorithms, the “mean from list 1” option was chosen to calculate mean values of the individual jICA-derived network maps (including HC, rCD, and aCD data) showing group differences in the ANCOVA models. These mean maps were subsequently subject of Spearman correlations with Z-transformed PET/SPECT-based neurotransmitter/receptor maps. Adjustment for spatial autocorrelation was performed and 10000 permutations were used during computation of exact p-values. Statistical inference was based on nominal significance level of p < 0.05, followed by Bonferroni-correction.

**References**

1. Wang P, Yang J, Yin Z, Duan J, Zhang R, Sun J, et al. Amplitude of low-frequency fluctuation (ALFF) may be associated with cognitive impairment in schizophrenia: a correlation study. BMC psychiatry. 2019;19(1):30.

2. Zang YF, He Y, Zhu CZ, Cao QJ, Sui MQ, Liang M, et al. Altered baseline brain activity in children with ADHD revealed by resting-state functional MRI. Brain Dev. 2007;29(2):83-91.

3. Wolf RC, Werler F, Schmitgen MM, Wolf ND, Wittemann M, Reith W, et al. Functional correlates of neurological soft signs in heavy cannabis users. Addict Biol. 2023;28(3):e13270.

4. Hirjak D, Rashidi M, Fritze S, Bertolino AL, Geiger LS, Zang Z, et al. Patterns of co-altered brain structure and function underlying neurological soft signs in schizophrenia spectrum disorders. Hum Brain Mapp. 2019;40(17):5029-41.

5. Zang Y, Jiang T, Lu Y, He Y, Tian L. Regional homogeneity approach to fMRI data analysis. Neuroimage. 2004;22(1):394-400.

6. Jiang L, Zuo XN. Regional Homogeneity: A Multimodal, Multiscale Neuroimaging Marker of the Human Connectome. Neuroscientist. 2016;22(5):486-505.

7. Sui J, Adali T, Yu Q, Chen J, Calhoun VD. A review of multivariate methods for multimodal fusion of brain imaging data. J Neurosci Methods. 2012;204(1):68-81.

8. Calhoun VD, Liu J, Adali T. A review of group ICA for fMRI data and ICA for joint inference of imaging, genetic, and ERP data. Neuroimage. 2009;45(1 Suppl):S163-72.

9. Ramezani M, Abolmaesumi P, Marble K, Trang H, Johnsrude I. Fusion analysis of functional MRI data for classification of individuals based on patterns of activation. Brain Imaging Behav. 2015;9(2):149-61.

10. Sui J, Adali T, Pearlson GD, Clark VP, Calhoun VD. A method for accurate group difference detection by constraining the mixing coefficients in an ICA framework. Hum Brain Mapp. 2009;30(9):2953-70.

11. Calhoun VD, Adali T, Kiehl KA, Astur R, Pekar JJ, Pearlson GD. A method for multitask fMRI data fusion applied to schizophrenia. Hum Brain Mapp. 2006;27(7):598-610.

12. Liu J, Pearlson G, Windemuth A, Ruano G, Perrone-Bizzozero NI, Calhoun V. Combining fMRI and SNP data to investigate connections between brain function and genetics using parallel ICA. Hum Brain Mapp. 2009;30(1):241-55.

13. Liu J, Kiehl KA, Pearlson G, Perrone-Bizzozero NI, Eichele T, Calhoun VD. Genetic determinants of target and novelty-related event-related potentials in the auditory oddball response. Neuroimage. 2009;46(3):809-16.

14. Himberg J, Hyvarinen A, Esposito F. Validating the independent components of neuroimaging time series via clustering and visualization. Neuroimage. 2004;22(3):1214-22.

15. Dukart J, Holiga S, Rullmann M, Lanzenberger R, Hawkins PCT, Mehta MA, et al. JuSpace: A tool for spatial correlation analyses of magnetic resonance imaging data with nuclear imaging derived neurotransmitter maps. Hum Brain Mapp. 2021;42(3):555-66.
